# Investigating the role of *Lactococcus lactis* D1813, salinity, and dissolved oxygen on the nutritional, chromatic, and textural profile of *Litopenaeus vannamei*

**DOI:** 10.1016/j.fochx.2025.102404

**Published:** 2025-03-19

**Authors:** Muhammad Adil, Guo Xinbo, Junpeng Cai, Muhammad Waseem, Muhammad Faisal Manzoor, Crossby Osei Tutu

**Affiliations:** a School of Food Science and Engineering, South China University of Technology, Guangzhou 510641, China; b Department of Food Science & Technology, Faculty of Agriculture & Environment, The Islamia University of Bahawalpur, Punjab, 63100, Pakistan; c Guangdong Provincial Key Laboratory of Intelligent Food Manufacturing, School of Food Science and Engineering, Foshan University, Foshan, China; d Faculty of Sciences and Technology, ILMA University, Karachi, Pakistan; e Department of Family and Consumer Sciences, University of Ghana, Accra, Ghana

**Keywords:** Aquaculture, Shrimps, Probiotics, Metabolomics, Nutrients, Vitamins

## Abstract

The present study investigated the effect of *L. lactis* D1813, salinity (8 and 25 ppt), dissolved oxygen (8.5 and 3.5 mg/L), and freshwater shrimp reared in natural freshwater conditions (0 ppt salinity, ∼7.5 mg/L DO) without probiotic addition on its nutritional, chromatic and textural profile. Among the nutritional composition results, the highest protein, ash, and fiber contents were observed in the Huang group meat, i.e., 15.25, 4.50, and 3.99 g/100 g, respectively. Likewise, the results for the analysis of the mineral anticipated that the highest levels of K, Cu, Se, Fe, and Zn were found in Huang head group, i.e., 536, 6.3, 603, 9.2 and 4.6 mg/kg, respectively with high levels of astaxanthin, i.e., 270 μg/g. The Huang meat group also observed maximum chewiness and cohesiveness, i.e., 79.5 and 0.4, respectively. Furthermore, the results for amino acids elucidated the presence of the highest concentrations in the Huang meat group, such as isoleucine, valine, threonine, methionine, and arginine, i.e., 1.85, 1.33, 1.17, 1.44, and 1.33 g/100 g, respectively when compared with control. The highest levels of polyunsaturated fatty acids, such as eicosapentaenoic acid and docosahexaenoic acid, were observed in the Huang head group, i.e., 11.29 and 5.57 g/100 g. Our findings suggest that using L. *lactis* D1813 as probiotics along with the salinity and dissolved oxygen of 8 ppt and 7.5 mg/L significantly improves the nutritional profile of L. *vannamei* with better nutrient efficiencies. Furthermore, the study suggests probing the different probiotics in shrimp physiology, such as immune response and disease resistance.

## Highlights

• *Litopenaeus vannamei* is well recognized for its valuable nutrition and health benefits.
• Adding *Lactococcus lactis* in L. *vannamei* enhances the nutritional profile of shrimp.
• Salinity & dissolved oxygen of 8 ppt & 8.5 mg/L improves astaxanthins & texture.
• High Salinity of 25 ppt & low dissolved oxygen of 3.5 mg/L affect amino & fatty acids.

## 1 Introduction

Pacific white shrimp *(Litopenaeus vannamei)* is among the most significant seafood traded worldwide due to its promising nutrient potential (Figueredo et al., 2023). Globally, *L. vannamei* is highly preferred for aquaculture farming owing to its fast growth and development and wide adaptability to different farming environments (Wyban, 2019; Adil et al., 2025). Global statistical data reports farming in over forty-seven countries with a total production of 6.82 million tons. Among these, China ranks first with an annual production of 2.09 million metric tons, followed by India, Ecuador, Indonesia, Vietnam, Thailand, Mexico, and Brazil (Susanto, 2021). *L. vannamei* are excellent sources of high-quality proteins (i.e., essential amino acids), low saturated with essential fatty acids, essential vitamins (i.e., cobalamin, niacin, tocopherol, and riboflavin), and minerals i.e., Se, Mg, Cu, I, P and Zn (Arshad et al., 2022). The nutritional composition of L. *vannamei* reveals that it contains 65–70 % phospholipids, 15–20 % cholesterol, 10–20 % total acylglycerol, 32 % polyunsaturated fatty acids (PUFA) (i.e., ω-3PUFA 64 %, and ω-6PUFA 33 %) and astaxanthin and each lipid type (phospholipids, cholesterol, acylglycerol, and PUFAs) constitutes a portion of the total fatty acids or lipids present in the shrimp.(Guo et al., 2024; Tu et al., 2022). *L. lactis* D1813 is a well-researched probiotic strain (Pan, 2019) recognized for its positive impact on gut health, immune system support, and metabolic enhancements in aquatic species (Cano-Lozano et al., 2022; Adil et al., 2025). Dissolved oxygen (DO) and salinity are vital water quality parameters in shrimp aquaculture, significantly affecting growth, survival, and overall health (Cano-Lozano et al., 2022). Shrimps can tolerate salinity levels of 5 to 35 %, with an optimal range of 15 to 25 % for better survival and growth. Maintaining dissolved oxygen (DO) levels above 5 mg/L is essential for optimal cultivating L. *vannamei*, as levels below 2 mg/L can significantly hinder their growth (Jaffer et al., 2020).

Recent studies have reported the positive role of probiotics in improving the digestion and absorption of proteins and amino acids by modifying the metabolic pathways of host cells and breaking them down into simpler, more readily absorbable components (Adil et al., 2025; Yan et al., 2020). Also, probiotics are known to play a pivotal role in improving bioactive compounds such as vitamins B_12_ and biotin, essential amino acids, protein metabolisms, growth performances, and survival rates by enhancing the bioavailability of these nutrients (Xie et al., 2019; Osei Tutu et al., 2024, Osei Tutu et al., 2023, Akonor, Osei Tutu, Affrifah, Budu, & Saalia, 2023, Osei Tutu, Amissah, Amissah, & Saalia, 2019). Similarly, adding probiotics in *Penaeus monodon* (Giant Tiger Prawn/Black Tiger Shrimp) enhanced the nutritional quality, texture, protein digestibility, and color profile Pinho and Emerenciano (2021).

*Lates calcarifer* (Asian sea bass) inoculation with *Lactobacillus plantarum* at salinity 10 ppt and 7.8 mg/L dissolved oxygen levels significantly increased muscle protein content compared to the control group. Kewcharoen and Srisapoome (2019) showed that L. *vannamei* supplemented with *Bacillus subtilis* AQAHBS001 with 5 mg/L dissolved oxygen increased amino acid concentrations. Also, Zhuo et al. (2021) reported that *L.vannamei* supplemented with probiotics at a salinity of 11 ppt and dissolved oxygen levels of 5.5 mg/L resulted in increased levels of polyunsaturated fatty acids. Likewise, Seenivasan et al. (2012) explained that *Lactobacillus sporogenes* supplementation in the diet of freshwater prawn (*Macrobrachium rosenbergii)* post-larvae (PL) resulted in improved survival, growth, and biochemical composition when maintained at a dissolved oxygen level of 7.5 mg/L, which falls within the typical range for freshwater environments (7–7.5 mg/L). Despite extensive research on shrimp health, growth, nutrition, and environmental factors, a notable gap exists in evaluating the combined effect of probiotics with different salinity and dissolved oxygen levels on the nutritional profile along with better color and texture characteristics. Therefore, the present study was planned to investigate the role of L. *lactis* D1813, salinity, and dissolved oxygen level on nutritional, chromatic and textural Profile of L. *vannamei.*

## 2 Materials and methods

### 2.1 Materials, chemicals and reagents

Fresh and healthy shrimp samples (250 specimens of each group averaging a weight of 23 g each) were obtained from domestic farms in Hunan Province, China, and stored in ice bags at 4 ± 1 °C to maintain their freshness and nutritional quality. The chemicals and reagents, including methanol, acetonitrile, ethanol, chloroform, isopropanol, hexane, hydrochloric acid, sodium hydroxide, ammonium acetate, formic acid, and acetic acid, were all of the analytical grade and sourced from Fisher Chemicals (Saint Louis, MO, USA). The probiotic strains of L. *lactis* D1813 were obtained from Symbiotic Biotechnology Co., Ltd., Beijing, China.

### 2.2 Experimental diet and inoculum of L. *lactis* D1813

The experimental diet of *L.vannamei* is shown in Table 1. All dry ingredients were carefully measured and combined. Distilled water (410 mL/kg) was added to the dry mix, and gelatin (40 g/kg) served as a binder (Valdez-Chavez et al., 2024; Waseem et al., 2024, Akonor et al., 2022). The mixture was then processed through a meat grinder fitted with a die 1.2 mm diameter. The resulting wet strings were allowed to dry at room temperature for 24 h. Once dried, the diets were broken down into appropriately sized pellets. They were stored at −20 °C until it was time to feed. The proximate analysis was performed following AOAC methods (AOAC 1992). The inoculation of L. *lactis* D1813 followed the standard protocol described by Liu et al. (2022). A precisely measured 0.01 g/L of *L. lactis* D1813 was added to the rearing water of shrimps twice a day along with the live feeds (rotifers, i.e., *Brachionus plicatilis*, enriched with microalgae species *Nannochloropsis* and *Isochrysis*) 15 min before feeding the shrimps. The mixture was then gently aerated by a fine bubble diffuser (Aqua Air 8000 Saint Louis, MO, USA, 8-in. diameter, producing bubbles of 20–100 μm) to ensure uniform distribution of the inoculum and allowed to rest for a few minutes. The final volume of L. *lactis* D1813 in the water was adjusted at 10^6^ CFU/mL. This process was repeated twice a day to maintain consistent probiotics (i.e., *L. lactis* D1813) exposures.

**Table 1:** Ingredient (g/kg dry matter) and proximate composition of basal and experimental diets.

Ingredient	Quantity (g/kg)
Fish meal	254
Soybean meal	320
Wheat flour	271
Shrimp shell meal	30
Wheat gluten	48
Fish oil	30
Lecithin	10
Vitamin complex	20
Mineral complex	10
Proximate composition (g/kg dry weight)	
Dry matter	905
Crude protein	475.2
Crude lipid	109.6
Crude fiber	48.6
Crude ash	73.4

### 2.3 Experimental design and sample preparation

The L. *vannamei* samples inoculated with L. *lactis* D1813 were reared for one week before and divided into three experimental groups. Each group consisted of thirty shrimp, which were randomly selected and acclimatized to the experimental conditions: Huang (dissolved oxygen (DO) levels 8.5 ± 0.5 mg/L, salinity levels 8 ± 0.1 ppt), T3BS (dissolved oxygen levels 3.5 ± 0.5 mg/L, salinity levels 25 ± 0.1 ppt), and the Wei group, i.e., freshwater shrimp reared in natural freshwater conditions (0 ppt salinity, ∼7.5 mg/L DO) without probiotic addition. All the study groups were acclimatized to the designated salinity and dissolved oxygen conditions for one week. The experiment was conducted over seven weeks in 90 L of white plastic tanks equipped with full aeration systems to maintain the dissolved oxygen and uniform water circulation. During the entire experimental process, the water temperature for all the groups, including the Wei group (control), was adjusted at 26 ± 2 °C and dissolved oxygen levels at 8.5 ± 0.5, 3.5 ± 0.5 and 7.5 ± 0.5 mg/L for Huang, T3BS and Wei groups, respectively. Shrimp had free access to feed twice a day (at 9:00 and 17:00) and water *adlibitum*, with the water changed every five h, with approximately 20 % of the tank water being replaced each time to maintain water quality and ensure proper dissolved oxygen levels.

Additionally, the brine was continuously aerated every 24 h. Twenty-four hours before the experiments, the feeding of shrimps was stopped, and thirty shrimp were randomly selected from each group without exoskeleton (*n* = 30) for analysis. Separate shrimp samples were used for meat and head analysis to maintain accuracy in assessing the specific composition of each portion. For each test (such as nutritional composition, color, texture, minerals, etc.), three shrimp were used, and each parameter was tested in triplicate (n = 3). The experiment complied with the ethical protocol standard (No. L20090101). Muscle meat and head of shrimps without exoskeletons from each treatment group were subjected to liquid nitrogen flash freezing −196 °C (− 320.8 °F) and thereafter kept in an ultra-freezer (Thermo Scientific ULT1786–3-V40) at −80 ± 1 °C for further analyses in the study.

### 2.4 Nutritional composition of meat and head of Wei, Huang, and T3BS *L. vannamei*

Among the nutritional composition of muscle meat and head, moisture was determined using the oven-drying method (105 °C, for 24 h) by following the official method of the Association of Official Analytical Chemists (AOAC), i.e., AOAC method no. 950.46 as documented by Maisarah et al. (2014) protein by micro-Kjeldahl procedure, i.e., AOAC method no. 928.08, fat by Soxhlet extraction, i.e., AOAC method no. 991.36, ash by muffle furnace at 550 °C, i.e., AOAC method no. 920.153, fiber by organic residue remaining after acid and alkali digestion using sulfuric acid and sodium hydroxide. Carbohydrates were determined by NIFEXT fraction, using the equation according to the Tomita & dos Santos CARVALHO, 2021;

Carbohydrates = 100 - % (Moisture + protein + fat + ash + fiber).

### 2.5 Color determination of meat and head of Wei, Huang, and T3BS *L. vannamei*

Each sample of muscle meat and head of shrimp was tested for the *L*^⁎^ values (brightness), *a*^⁎^ values (redness), and *b*^⁎^ values (yellowness) using the NR60CP precision calorimeter (NR60CP, 3NH Technology Co., Ltd., China). The *a*^⁎^ and *b*^⁎^ values represent the difference in redness (+ = redder, − = greener) and yellowness (+ = yellower, − = bluer). Color measurements were performed with a D65 illuminant, an aperture size of 8 mm, and a 10° observation angle.

### 2.6 Texture determination of meat and head of Wei, Huang, and T3BS *L. vannamei*

Textural analysis of shrimp meat and the head of all treatment groups was performed using a TA.XT Plus Texture Analyzer (Stable Micro Systems, United Kingdom). The hardness, springiness, cohesiveness, chewiness, and adhesiveness of all samples were measured by compressing the double compression using the 36 mm probe at the testing parameters of pre-test speeds of 2.0 mm/s, test speeds of 1.0 mm/s, post-test speeds of 10.0 mm/s, distance of 6.0 mm, time of 5 s, load cell weight of 50 kg and force of 10 *g*.

### 2.7 Minerals determination of meat and head of Wei, Huang, and T3BS *L. vannamei*

The mineral contents were assessed using the inductively coupled plasma emission spectrometer (ICAP7400; Thermo Electron, Massachusetts, MA, USA). Precisely measured 4.0 g of each muscle meat and head sample was placed in a polytetrafluoroethylene (PTFE) tube, and then 12 mL of nitric acid (i.e., Conc. ∼ 68 %) was added in it for digestion (Beijing Chemical Works, China,). The process of digestion was prolonged until the sample became colorless/transparent. Once the solution had cooled down, it was moved to a 50 mL volumetric flask and diluted to a precise volume using double-deionized water. Samples and reagent blanks were tested against standards for the respective minerals (i.e., Na, K, Mg, Ca, P, Fe, Zn, Cu, and Se).

### 2.8 Astaxanthin determination of meat and head of Wei, Huang, and T3BS *L. vannamei*

The extraction of astaxanthin was carried out following the method described by Hu et al. (2019). A quantity of 200 mg of sample material was placed into a centrifuge tube with a volume of 50 mL. Afterward, the sample was mixed in an accurate volume of 5 mL of dichloromethane and methanol (1:3 ratio, *v/v*) solvent mixture. The mixture was then treated with oscillation using a SHY-2 oscillator (Putian Technologies in Changzhou, Suzhou, China) for 3 h and centrifuged at 5000 rpm for 15 min at 4 °C. The precipitates were subjected to an additional step where a mixture of dichloromethane and methanol (1,3 v/v) was added, along with the supernatant. The samples were collected, and an equivalent quantity of petroleum ether was added and mixed. The petroleum ether layer was subjected to nitrogen purging using MGS-2200H equipment (EYELLA business, Tokyo, Japan) for 30 min to eliminate organic solvents and extract astaxanthin. The dehydrated astaxanthin was dissolved in 5 mL of n-hexane and filtered using a 0.45 μm membrane filter to eliminate any remaining solid particles. Astaxanthin was determined using C_18_ (4.6 mm × 250 mm × 5 μm, Agilent Technologies, Santa Clara, CA, USA) column at high-performance liquid chromatography (HPLC) with an e2695 instrument (Milford, MA, Walters, USA) using methanol and ultrapure water as mobile phase, sample injection of 10 μL, flow rate of 1 mL/min, column temperature of 35 °C and at 480 nm.

### 2.9 Amino acids determination of meat and head of Wei, Huang, and T3BS *L. vannamei*

The identification and quantification of amino acids were carried out using an automated amino acid analyzer (L-8900; Hitachi, Tokyo, Japan) per the protocol outlined by Wu et al. (2020). A precise 0.5 g of each sample was combined with 10 mL of concentrated hydrochloric acid (∼6 mol/L) in sample tubes, which were then hermetically sealed. Nitrogen gas was introduced into the sealed tubes, and the samples were allowed to react for 8 h at 100 °C. After this reaction, the volume was adjusted to 100 mL with distilled water. The resulting mixture was extracted by vacuum freeze-drying down to 1 mL, which was subsequently mixed with 2 mL of diluted hydrochloric acid (∼0.02 mol/L) and filtered through a 0.22 μm filter. A 20 μL aliquot of the filtrate was injected into the automated amino acid analyzer. The amino acids were identified and quantified using standard methods and internal standards. The concentration of amino acids was reported as g/100 g of the sample.

### 2.10 Fatty acids determination of meat and head of Wei, Huang, and T3BS *L. vannamei*

Fats were extracted from the samples using a chloroform-methanol solution (2:1, *v*/v) per the method outlined by Das et al. (2021) to determine fatty acids. The extracted fats were saponified with potassium hydroxide in methanol and converted into fatty acid methyl esters by reacting with sulfuric acid-methanol solution (12.5 %, *w/v*). The fatty acid methyl esters were extracted using n-hexane and analyzed on gas chromatography–mass spectrometry using a Shimadzu GCMS-TQ8050NX (Shimadzu, Tokyo, Japan) equipped with DB-23 column (60 m × 0.25 mm × 0.25 μm, Agilent Technologies, Santa Clara, CA, USA). Helium was used as the carrier of gas at a flow rate of 1.0 mL/min, with a split ratio of 1:5. The initial column temperature was set at 120 °C for 5 min, followed by an increase up to 240 °C at a rate of 4 °C/min and held at 240 °C for 10 min. The temperature was then raised to 250 °C at a rate of 5 °C/min and held at 250 °C for 25 min. The injector temperature was maintained at 280 °C, and the ion source temperature was set to 250 °C. A sample volume of 1 μL was injected. Fatty acid methyl esters were identified by comparing the retention times with those of a fatty acid methyl ester standard mixture (Supelco 37 Component FAME Mix; Supelco Inc., Bellefonte, PA, USA). The fatty acid concentrations were quantified and expressed as g/100 g of total fatty acids identified.

### 2.11 Statistical analysis

All analyses, including the nutritional composition, textural profile, color profile, and fatty acid profiles, were performed in triplicates (*n* = 3) with means ± standard deviation (S.D.) and assessed for significance using one-way ANOVA followed by Duncan's multiple range tests at Statistical Program for Social Sciences 13.0 (SPSS Inc., Chicago, USA, v.13.0). The level of significance for all analysis samples was set at *p* < 0.05.

## 3 Results and discussion

### 3.1 Nutritional composition of meat and head of Wei, Huang, and T3BS *L. vannamei*

The highest protein content was observed in the Huang meat group, i.e., 15.24 g/100 g, followed by the T3BS group and the Wei group, i.e., 13.79 and 12.65 g/100 g, respectively. However, the highest fat contents found in the Huang head group exhibited the highest value, i.e., 4.18 g/100 g, compared to T3BS and Wei, i.e., 3.77 and 3.78 g/100 g. Similarly, the Huang head group demonstrated the highest levels of ash and fiber, i.e., 6.44 and 5.86, when compared with T3BS and Wei, i.e., 4.92, 4.89, 4.22, and 4.45 g/100 g., respectively (Table 2). Goh et al. (2023) explained that one approach to improving nutrition is the supplementation of probiotics in feed. This can enhance digestibility and efficiency, ensuring that vannamei shrimp absorb the feed effectively, ultimately leading to optimal growth in the shrimp. Herdianti et al. (2015) noted that bacteria present in probiotics help break down toxic chemical compounds and speed up the nutrient cycling process, which in turn helps manage disease outbreaks. Hasim et al. (2021) found that adding probiotics to feed significantly boosts shrimp's growth rate and production. Likewise, Zheng et al. (2017) reported that supplementing L. *vannamei* with *Lactobacillus plantarum* at 10 ppt salinity and 7.8 mg/L dissolved oxygen levels significantly enhanced the muscle proteins. However, Escamilla-Montes et al. (2023) elucidated that *L.Lactis* D1813 probiotic inoculation in *L.vannamei* 27 ppt salinity and 1.8 mg/L dissolved oxygen levels lowered the muscle proteins. Jannathulla et al. (2019) found that *P. vannamei* reared at 25 ppt had lower crude fat (5.66 g/kg) than those exposed to salinity of 6 ppt (6.08 g/kg). Similary, Supriatna et al. (2017) explained that dissolved oxygen (DO) levels significantly impact shrimp nutrition, with higher DO (8 mg/L) enhancing nutrient uptake and growth, while lower DO (2.5 mg/L) tends to reduce metabolic activity and nutrient absorption in shrimp. Also, Silva et al. (2010) reported that the addition of *Penaeus monodon* along with *Bifidobacterium bifidum* at 25 ppt salinity and 2.2 mg/L dissolved oxygen levels decreased the ash contents, i.e., 3.5 g/100 g, when compared with the freshwater *Penaeus monodon,* i.e., 4.1 g/100. Likewise, Qin et al. (2024) showed the *Farfantepenaeus subtilis* at 9.8 ppt salinity in shrimps resulted in an increase of fiber contents in shrimp head, i.e., 78 g/100 g when compared to 30 ppt salinity, i.e., 74 g/100 g, respectively. Simlilarly, Mozanzadeh et al. (2023) explained that inoculation of Asian Sea bass *(Lates calcarifer)* with *Lactobacillus acidophilus* at high dissolve oxygen (˃7.5 mg/L) showed higher fiber contents of 81 g/100 g when compared with fiber contents of low dissolved oxygen (˂2.5 mg/L), i.e., 77.6 g/100 g, respectively. Moreover, Perez-Velazquez et al. (2007) explain that shrimp reared at 2 ppt salinity exhibited higher final weight and weight gain than those at 35 and 50 ppt, with significantly lower feed conversion ratio (FCR) at 2 ppt.

**Table 2:** Nutritional composition of L. *vannamei* shrimp meat and head (g/100 g).

Parameters	Huang meat	Wei meat	T3BS meat	Huang head	Wei head	T3BS head
Protein	15.24 ± 0.11^a^	12.65 ± 0.22^c^	13.78 ± 0.01^b^	8.66 ± 1.02^d^	8.44 ± 1.09^de^	8.67 ± 1.02^d^
Fat	0.74 ± 0.03^c^	0.75 ± 0.03^c^	0.75 ± 0.03^c^	4.18 ± 0.01^a^	3.78 ± 0.01^b^	3.77 ± 0.21^b^
Ash	1.94 ± 0.17^c^	1.93 ± 0.17^c^	1.93 ± 0.17^c^	6.44 ± 2.43^a^	4.89 ± 1.83^b^	4.92 ± 1.81^b^
Fiber	0.29 ± 0.02^c^	0.27 ± 0.01^c^	0.28 ± 0.01^c^	5.86 ± 1.21^a^	4.45 ± 1.32^b^	4.22 ± 1.55^bc^
Carbohydrates	8.46 ± 0.23^b^	8.47 ± 0.24^b^	8.47 ± 0.23^b^	10.5 ± 1.06^a^	10.48 ± 1.07^a^	10.5 ± 1.06^a^
Moisture	74.69 ± 1.06^a^	74.7 ± 1.04^a^	74.71 ± 1.03^a^	72.71 ± 0.04^b^	72.48 ± 1.18^bc^	72.55 ± 0.69^b^

Results are present as means ± SD and *n* = 3. Values with different alphabets are significantly (*p* *<* *0.05*) different among rows.

### 3.2 Minerals profile of meat and head of Wei, Huang, and T3BS *L. vannamei*

The results for the mineral composition of different groups of L. *vannamei* showed the presence of the highest levels of K, Mg, P, Cu, Se, Fe, and Zn in the Huang head group, i.e., 536, 187, 833, 6, 603, 9, and 4.6 mg/kg, followed by the T3BS group, i.e., 2.4, 64.5, 504, 1., 362, 4.3, and 3.1 mg/kg, and Wei group i.e., 4.4, 60.8, 447, 1.8, 337, 6.5, and 3.6 mg/kg, respectively (Table 3). Madhana et al. (2021) explained that *Lactobacillus* strains have been shown to influence gut microbiota, enhancing the efficiency of mineral absorption in the digestive system of L. *vannamei*. Likewise, Goh et al. (2023) explained that adding probiotics enhances shrimp's mineral profile by influencing gut microbiota, leading to better digestion and absorption of essential minerals from their diet. Similarly, Wang et al. (2022) reported that the addition of *Penaeus monodon* feed with the *Lactobacillus* at 8.5–10 ppt salinity and 7.5–9.8 mg/L dissolved oxygen levels significantly (*p* < 0.05) higher concentrations of K, Se and P levels, i.e., 436, 601 and 549 mg/kg when compared at 30–35 ppt salinity and 1.2–3.2 mg/L dissolved oxygen levels which showed slightly lower magnitudes of these minerals as; 413, 548 and 498 mg/kg, respectively. In this nexus, Jannathulla et al. (2017) examined how water salinity levels (ranging from 3 to 60 ppt) affect the growth and overall mineral composition of *P. vannamei* shrimp. The findings showed that shrimp raised in very low (3 ppt) and very high (60 ppt) salinities experienced significant adverse effects on growth and mineral deposition. However, Tran-Ngoc et al. (2016) explained that Nile tilapia (*Oreochromis niloticus*), with low dissolved oxygen levels in shrimp, remain under stress, disrupting their metabolic processes and hindering their ability to absorb minerals, which can result in reduced mineral levels in their tissues. Likewise, another study by Kane and Sinclair (2018) also showed improved concentrations of Zn, Cu, and Fe, i.e., 4.6, 6.2, and 9.3 mg/kg in starfish with *Lactobacillus rhamnosus* at high salinity levels of ˃20 ppt. However, the lower salinity group (i.e., ˂3.2 ppt) showed lower magnitudes of these minerals, i.e., 3, 6, and 7.5 mg/kg. Also, Manikandan and Felix (2021) delineated that higher salt levels (˃35 %) and dissolved oxygen (i.e., 1.5 mg/L) decrease Mg, P and K contents, i.e., 211, 187, and 432 when compared with lower salinity (8 ppt) and higher dissolved oxygen levels (i.e., 7.5 mg/L) and elucidated improved mineral elements i.e., 233, 198 and 512 mg/kg, respectively. Likewise, Manikandan and Felix (2021) explained that *Saccharomyces boulardii* inoculation with *Amblyrhynchus cristatus* at low salinity (4 ppt) decreased the Zn, Fe, and Cu concentrations, i.e., 2.9, 7.2, and 6.8 mg/kg, respectively. Similarly, *Lactobacillus, Bifidobacterium* and *Saccharomyces boulardii* improved the Mg, K, Se and Zn concentrations from 131 to 144, 419–433, 574–605 and 4.9–6.4 mg/kg at 1.5–8 mg/L dissolved oxygen (Galkanda-Arachchige et al., 2021; Moura et al., 2023; Haider et al., 2025, Akonor, Osei Tutu, Affrifah, Budu, & Saalia, 2023, Asiedu et al., 2025). Tran-Ngoc et al. (2016) explained that the supplementation of probiotics enhances shrimp culture performance, including growth, survival, and total biomass, which provides essential inorganic substances like trace minerals (Ca, Mg, Zn, and Fe) that support shrimp growth.

**Table 3:** Mineral contents of L. *vannamei* shrimp meat and head (mg kg^− 1^).

Minerals	Huang meat	Wei meat	T3BS meat	Huang head	Wei head	T3BS head
Na	482.1 ± 2^d^	287.57 ± 3^e^	493.7 ± 4^c^	490.4 ± 5^cd^	536.11 ± 6^b^	543.62 ± 5^a^
K	4.09 ± 0.12^c^	4.40 ± 0.08^b^	2.36 ± 0.04^e^	5.36 ± 0.09^a^	3.84 ± 0.09^d^	1.47 ± 0.07^f^
Mg	76.6 ± 0.18^cd^	60.8 ± 0.21^e^	65.79 ± 1.15^d^	187.46 ± 1.3^a^	105.80 ± 1.5^b^	78.05 ± 1.15^c^
Ca	80.9 ± 0.21^f^	162.5 ± 0.3^b^	110.8 ± 0.4^e^	136.63 ± 1.6^d^	185.26 ± 1.7^a^	146.05 ± 2.1^c^
P	632.05 ± 3^b^	447.5 ± 3^f^	504.6 ± 5^d^	833.52 ± 6^a^	485.09 ± 3^e^	525.05 ± 4^c^
Fe	5.61 ± 0.12^d^	6.53 ± 0.12^c^	4.27 ± 0.08^e^	9.19 ± 0.09^a^	7.18 ± 0.13^b^	6.45 ± 0.21^c^
Zn	4.64 ± 0.11^b^	3.62 ± 0.07^e^	3.12 ± 0.05^f^	5.03 ± 0.34^a^	4.05 ± 0.22^d^	4.38 ± 0.38^c^
Cu	2.09 ± 0.08^c^	1.86 ± 0.02^d^	1.79 ± 0.02^d^	6.24 ± 0.11^a^	6.17 ± 0.23^a^	3.03 ± 0.11^b^
Se	603.4 ± 5^b^	336.8 ± 3^f^	358.8 ± 3^e^	677.75 ± 5^a^	509.95 ± 5^c^	404.21 ± 6^d^

Results are present as means ± SD and n = 3. Values with different alphabets are significantly (*p* *<* *0.05*) different among rows.

### 3.3 Astaxanthin and chromatic profile of meat and head of Wei, Huang, and T3BS *L. vannamei*

**Fig. 1:**
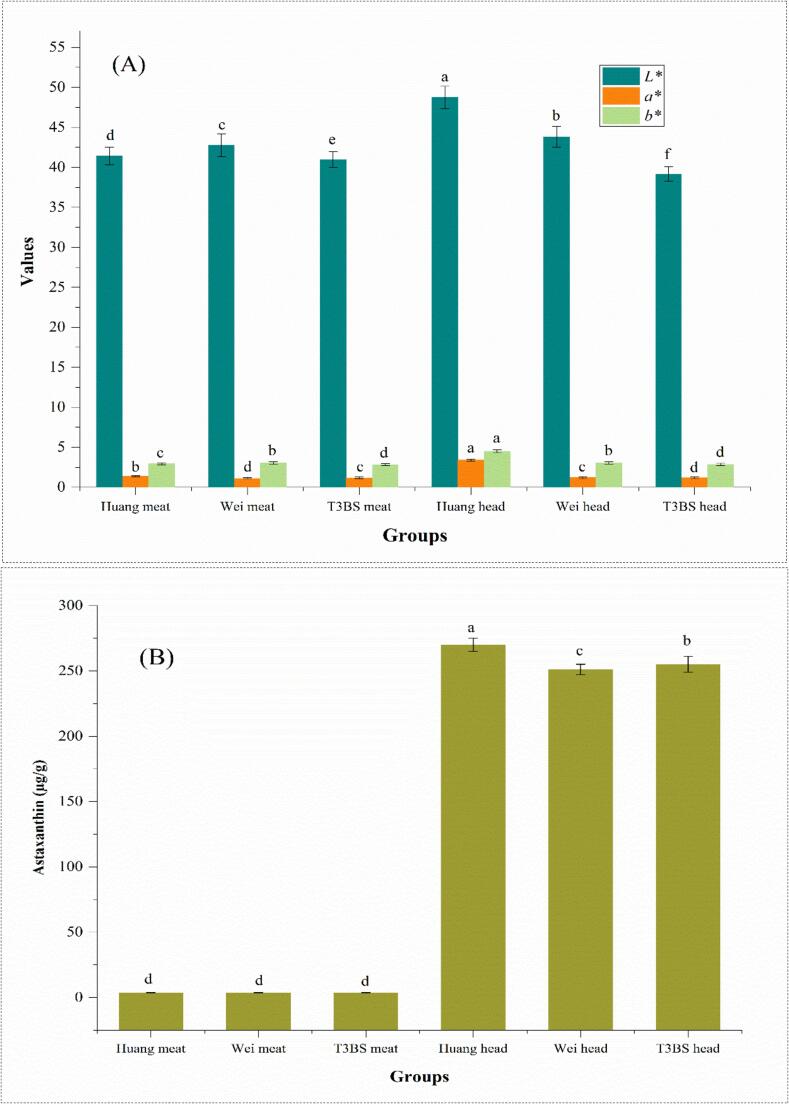
Chromatic profile (A) and astaxanthin (B) of meat and head of Wei, Huang and T3BS *L. vannamei.* Results are present as means ± SD. Values with different alphabets are significantly (*p* *<* *0.05*) different.

The highest levels of astaxanthin were observed in the Huang head group, i.e., 270 μg/g, followed by T3BS and Wei, i.e., 255 and 251.04 μg/g, respectively (Fig. 1A). Astaxanthin is a pinkish-orange carotenoid that is soluble in fats, commonly found in seafood such as crustaceans, shellfish, crabs, shrimp, and fish, as well as in algae and certain plants Stachowiak and Szulc (2021). Astaxanthin exhibits antioxidant activity that surpasses that of β-carotene, vitamin C, vitamin E, lutein, lycopene, and various catechins, and astaxanthin antioxidant power is 100 to 500 times greater than that of α-tocopherol and 5 to 15 times more potent than other carotenoids Brendler and Williamson (2019). A study by Tran-Ngoc et al. (2016) explained that probiotics may improve overall shrimp health, supporting enzymatic activities that increase astaxanthin synthesis and deposition, particularly in the exoskeleton and muscle. Likewise, Nguyen et al. (2020) explained that probiotics, specially bacillus, modulate the gut microbiota and improve digestion; probiotics help in better absorption of carotenoids, leading to increased astaxanthin content in shrimp tissues, particularly in the muscle and exoskeleton. Likewise, Castex et al. (2009) showed comparable findings for the astaxanthin contents (i.e., 268 μg/g) in the head of *Litopenaeus stylirostris*on supplementing with the *Pediococcus acidilactici* at moderate salinity (9 ppt) and high dissolved oxygen (7.6 mg/L) when compared with freshwater *Litopenaeus stylirostris*on without probiotic inoculation i.e., 251 μg/g. Moreover, Gupta and Gupta (2014) explained that *Penaeus vannamei* and *Penaeus monodon* supplemented with *Bacillus cereus* under salinity conditions of (12–30 ppt) showed notable magnitudes of astaxanthin contents in the head form 254–260 μg/g. The results for the chromatic profile of L. *vannamei,* showed the highest levels of *L*^⁎^, *a*^⁎^ and *b*^⁎^ in the Huang head group, i.e., 48.73, 3.4, and 4.5 followed by T3BS head group (39.16, 1.2, & 2.85) and Wei head group (i.e., 43.8, 1.2 and 3.05), respectively (Fig. 1B). Luis-Villaseñor et al. (2012) explained that Shrimp blood agar plates with a rose-red color suggested the presence of healthy hemocytes, whereas hemolytic bacteria produced clear zones surrounding the colonies. Probiotic *Bacillus* strains were found to enhance shrimp health, which may also improve their coloration. In this nexus, Dawood et al. (2017) delineated that supplementing red sea bream *(Pagrus major)* with *Lactobacillus rhamnosus* at moderate levels of salinity (i.e., 9.5 ppt) and high dissolved oxygen (i.e., 6 mg/L) resulted in improved *L*^⁎^ and *a*^⁎^ values in the shrimp head, i.e., 47 and 0.5, while in other groups where salinity level of 2 ppt and dissolved oxygen of 1.5 mg/L showed significant lower *L*^⁎^ and *a*^⁎^ values, i.e., 41.5 and 0.4. Also, Chiu et al. (2021) portrayed that *Penaeus monodon* supplemented with *Bifidobacterium animalis* under high salinity levels (i.e., 28 ppt) and low dissolved oxygen (i.e., 1.8 mg/L) exhibited lower mean values for *L*^⁎,^ i.e., 38.7, when compared the freshwater Asian tiger shrimp, i.e., 43. Likewise, Wang et al. (2020) reported lower *L*^⁎^ and *b*^⁎^ values of 39.8 and 2.1 at lower oxygen levels of ˂2 mg/L and higher *L*^⁎^ and *b*^⁎^ values of 41.8 and 3.2 at high oxygen levels of >10 mg/L on the addition of *Lactobacillus acidophilus* in juvenile *P. monodon.* Similiarly, Nguyen et al. (2022) showed that the shrimps exposed to the salinity levels of ˂16–35 ppt and dissolved oxygen levels of 7.5–8.5 mg/L showed the *L*^⁎^, *a*^⁎^ and *b*^⁎^ values of 22–35, 0.9–1.9 and 2.1–2.9, respectively.

### 3.4 Textural profile of meat and head of Wei, Huang, and T3BS *L. vannamei*

**Fig. 2:**
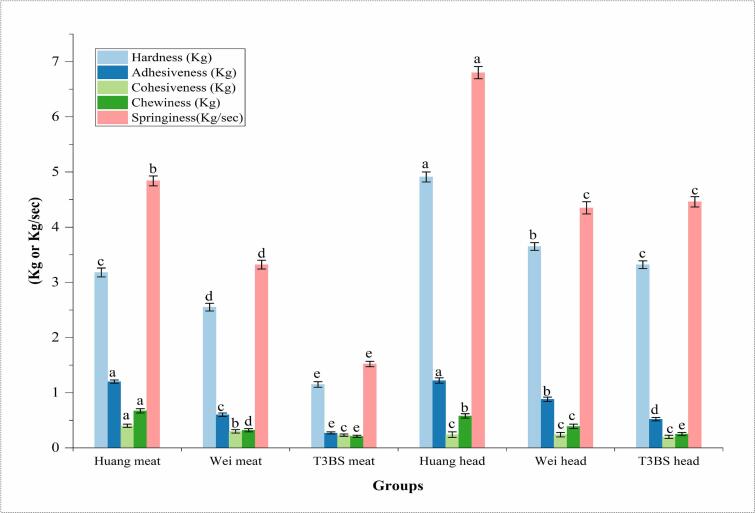
Textural profile of meat and head of Wei, Huang and T3BS *L. vannamei.* Results are present as means ± SD. Values with different alphabets are significantly (*p* *<* *0.05*) different.

Among the textural attributes of Wei, Huang and T3BS meat groups, the results anticipated significantly (*p* < 0.05) the highest values of hardness, chewiness, and springiness in the Huang meat group, i.e., 3.18, 0.67 kg, and 4.84 kg/s, followed by T3BS, i.e., 1.15, 0.21 kg, 1.52 kg/s and the Wei group i.e., 2.55, 0.32 kg, and 3.32 kg/s, respectively and highest springiness and hardness were also observed in the Huang head group, i.e., 4.91 kg, 6.8 kg/s, followed by T3BS and the Wei meat groups, i.e.,3.32, 3.65 kg, 4.46 and 4.35 kg/s, respectively (Fig. 2). Probiotics can positively influence gut health, enhance nutrient absorption, promote overall shrimp growth, and improve meat quality (Ajitha et al., 2004). Probiotic supplementation can positively impact muscle protein synthesis and reduce muscle degradation, resulting in firmer and more cohesive meat (Trabelsi et al., 2019; Suleman et al., 2025). Likewise, Cai et al. (2024) revealed that inoculation of fermented fish with Lactic acid bacteria at 2.3–8 mg/L dissolved oxygen showed measurable magnitudes of springiness cohesiveness, i.e., 0.23–0.39 and 0.8–0.9 kg/s. Also, Kurniaji et al. (2023) showed close linearity with our findings, wherein the researchers showed that L. *vannamei* supplementation with different probiotic sources at 35 ppt salinity, and low dissolved oxygen of 1.8 mg/L depicted lower values of hardness, chewiness, and cohesiveness, i.e., 5.8, 3.2 and 0.3 kg. Kamaruddin et al. (2021) reported that supplementing Sea worm *(Nereis sp)* with *Bacillus subtilis* at moderate salinity (2–6.5 ppt) and high dissolved oxygen (3.5–9 mg/L) resulted in increased hardness and chewiness in meat, i.e., 7.2–7.5 and 3.7–4.3 kg. High salinity significantly decreased myofibril diameter but increased myofibril density (Du et al., 2022). However, Hsieh et al. (2021) explained that shrimp may show reduced muscle growth and lower protein synthesis under low dissolved oxygen conditions. This can result in a decline in meat firmness, chewiness, and overall texture quality. High dissolved oxygen supports aerobic metabolism, which enhances muscle protein synthesis and decreases muscle degradation (Yang et al., 2024). In this nexus, Mareta et al. (2020) when *P. monodon* inoculated with *Lactobacillus acidophilus* at low oxygen levels (2 to 8 mg/L) decreases the hardness, chewiness, and cohesiveness, i.e., 7.6 to 7.5, 5.0 to 4.3, and 1.2 to 0.4 kg/s. Smiliarly, Roobab et al. (2020) demonstrated that the dissolved oxygen levels of 2.5 to 7.6 mg/L resulted in enhancement of hardness and chewiness of Atlantic shrimp from 6.5 to 7.0 and 3.9–4.3 kg, respectively.

### 3.5 Amino acids profile of meat and head of Wei, Huang, and T3BS *L. vannamei*

Among the amino acids profile of Wei, Huang and T3BS meat groups the results anticipated significantly (*p* < 0.05) the highest values of lysine, phenylalanine, leucine, isoleucine, valine, threonine, methionine and arginine in the Huang meat group, i.e., 1.88, 1.93, 1.96, 1.85, 1.33, 1.17, 1.44 and 1.33 g/100 g, followed by T3BS, i.e., 1.39, 1.28, 1.62, 1.41, 0.96, 0.90, 0.16 and 0.68 g/100 g and Wei group, i.e., 1.12, 1.26, 1.71, 1.60, 0.39, 1.74, 0.70 and 1.10 g/100 g, respectively (Table 4). Intestinal microbiota has a range of biological effects and aids in the digestion and absorption of proteins and amino acids (AAs) by breaking down complex subunits into simpler forms that are easier to absorb. This process can subsequently alter the metabolic pathways within host cells (Ringø et al., 2022). Probiotics enhance amino acids by expressing genes involved in l-Glutamine amino acid transport (Koyama et al., 2018). Another study by Ringø et al. (2022) demonstrated that inoculation of *Lactobacillus acidophilus* with *Penaeus monodon* at salinity 13–32 ppt and dissolved oxygen levels of 1.8–7.5 mg/L showed comparable magnitudes of glycine and threonine, i.e., 1.12–1.35 and 0.78–1.02 g/100 g. The decrease in amino acids of shrimps, together with high salinity, could be linked with osmotic stress, which impairs amino acid synthesis and absorption (Wafi et al., 2021; Suleman et al., 2025). Amino acids function as osmolytes, helping organisms adjust to environmental stress by managing extracellular solutions during their synthesis or breakdown (Zadehmohseni et al., 2020; Osei Tutu et al., 2024). When salinity levels rise, changes in amino acid content are noted as part of the organism's strategy to preserve osmotic balance, though these alterations can adversely affect overall nutritional quality (Li et al., 2017). Likewise, Toledo et al. (2019) reported that supplementation of *L. plantarum* with *Lates calcarifer* at 10–30 ppt salinity showed comparable concentrations of leucine and tyrosine, i.e., 2.02–1.65 and 1.78–1.45 g/100 g. Similarly, Goh et al. (2023) exhibited significant improvement in lysine and methionine levels from 1.4 to 1.94 and 0.92–1.45 g/100 g in *Penaeus vannamei* supplemented with L. *rhamnosus* at 8 ppt salinity and 7.2 mg/L dissolved oxygen. When dissolved oxygen levels are high, shrimp usually show increased metabolic activities, which include a boost in protein synthesis. It leads to higher levels of essential amino acids, promoting better growth, enhanced stress resistance, and improved nutritional quality (Nguyen et al., 2022). High oxygen levels facilitate aerobic respiration and ATP production, while low oxygen enhances the retention of amino acids, which could be associated with improved levels of amino acids (Glencross, 2021). Similarly, another study by Huynh et al. (2018) showed that inoculation of *Bacillus subtilis* with L. *vannamei* at 2.4–8.0 mg/L dissolved oxygen enhance amino acid concentrations such as glycine and threonine, i.e., 1.21–1.35 and 0.89–1.02 g/100 g. Likewise, Xie et al. (2019) indicated that high dissolved oxygen (˃8 mg/L) increased cellular respiration and amino acid synthesis while dissolved oxygen (˂3.5 mg/L) inhibit enzyme activities associated with protein synthesis.

**Table 4:** Amino acids profile of L. *vannamei* meat and head (g/100 g).

Amino acids	Huang meat	Wei meat	T3BS meat	Huang head	Wei head	T3BS head
Lysine	1.88 ± 0.03^a^	1.12 ± 0.03^c^	1.39 ± 0.03^b^	0.81 ± 0.03^e^	0.82 ± 0.04^de^	0.88 ± 0.02^d^
Phenylalanine	1.93 ± 0.04^a^	1.26 ± 0.02^b^	1.28 ± 0.02^b^	0.62 ± 0.02^c^	0.61 ± 0.02^c^	0.59 ± 0.03^c^
Leucine	1.96 ± 0.06^a^	1.71 ± 0.04^b^	1.62 ± 0.04^bc^	0.84 ± 0.04^c^	0.79 ± 0.03^cd^	0.81 ± 0.03^c^
Isoleucine	1.85 ± 0.05^a^	1.6 ± 0.03^b^	1.41 ± 0.03^c^	0.54 ± 0.02^d^	0.51 ± 0.02^d^	0.53 ± 0.02^d^
Valine	1.33 ± 0.02^a^	0.39 ± 0.02^e^	0.96 ± 0.02^b^	0.63 ± 0.03^c^	0.57 ± 0.03^d^	0.59 ± 0.03^cd^
Threonine	1.17 ± 0.03^a^	1.14 ± 0.05^a^	0.90 ± 0.03^b^	0.50 ± 0.02^c^	0.49 ± 0.02^c^	0.51 ± 0.02^c^
Methionine	1.44 ± 0.04^a^	0.7 ± 0.02^b^	0.16 ± 0.01^d^	0.58 ± 0.02^c^	0.57 ± 0.04^c^	0.58 ± 0.02^c^
Arginine	1.33 ± 0.01^a^	1.11 ± 0.03^b^	0.68 ± 0.03^d^	0.77 ± 0.04^cd^	0.78 ± 0.03^cd^	0.81 ± 0.03^c^
Histidine	0.71 ± 0.02^a^	0.48 ± 0.02^c^	0.58 ± 0.02^b^	0.29 ± 0.01^de^	0.33 ± 0.01^d^	0.31 ± 0.01^d^
Tyrosine	1.76 ± 0.05^a^	0.53 ± 0.04^b^	0.5 ± 0.02^b^	0.49 ± 0.03^bc^	0.52 ± 0.03^b^	0.48 ± 0.02^bc^
Alanine	1.16 ± 0.04^a^	0.66 ± 0.02^c^	0.13 ± 0.01^d^	0.83 ± 0.04^b^	0.85 ± 0.04^b^	0.81 ± 0.03^b^
Glycine	1.62 ± 0.03^a^	1.49 ± 0.01^b^	1.32 ± 0.04^c^	0.90 ± 0.05^de^	0.94 ± 0.03^d^	0.98 ± 0.04^d^
Proline	1.82 ± 0.02^a^	1.21 ± 0.02^c^	1.73 ± 0.04^b^	0.65 ± 0.03^d^	0.67 ± 0.02^d^	0.68 ± 0.03^d^
Glutamic Acid	1.88 ± 0.01^a^	1.51 ± 0.04^c^	1.74 ± 0.05^b^	1.72 ± 0.05^b^	1.71 ± 0.03^b^	1.72 ± 0.04^b^

Results are present as means ± SD and n = 3. Values with different alphabets are significantly (*p* *<* *0.05*) different among rows**.**

### 3.6 Fatty acids profile of meat and head of Wei, Huang, and T3BS *L. vannamei*

Among the fatty acids profile of Wei, Huang, and T3BS meat and head groups, the results anticipated significantly (*p* < 0.05) the highest levels of polyunsaturated fatty acids, i.e., C20:5n-3 eicosapentaenoic acid (EPA), C22:2n-6, and C22:6n-3 docosahexaenoic acid (DHA) in the Huang head group, i.e., 9.86 and 0.09 and 6.45 g/100 g followed by T3BS, i.e., 7.24, 0.05, 4.34 g/100 g and Wei head group, i.e., 3.09,0.06 and 4.91 g/100 g,respectively (Table 5). Dietary supplementation with probiotics can significantly influence the lipid metabolism in shrimp and modify the fatty acid profile (Ramezani-Fard et al., 2014). Probiotic supplementation in shrimp has been shown to affect the fatty acid profile, increasing the levels of polyunsaturated fatty acids (PUFAs) and essential lipids Yuhana and Zairin Jr (2017). The increase in the levels of essential fatty acids like EPA and DHA in shrimp tissues could be linked with the breakdown of dietary fats owing to the actions of probiotics (Yang et al., 2023). Likewise, Jannathulla et al. (2019) explained that shrimp in low salinity (2 ppt) were able to utilize fatty acids more effectively than those in high salinity (50 ppt), leading to improved growth and survival rates. It indicates that hypoosmotic stress (low salinity) boosts fatty acid utilization, whereas hyperosmotic stress (high salinity) restricts it, ultimately impacting shrimp health. Chen et al. (2020) demonstrated that *Dicentrarchus labrax* supplementation in *Lactobacillus acidophilus* at salinity levels of 14–30 ppt and dissolved oxygen levels of 2–7.5 mg/L showed measurable concentrations of EPA and DHA, i.e., 1.35–0.97 and 0.58–0.92 g/100 g. Low dissolved oxygen levels can disrupt the metabolic processes in shrimp, causing inefficient fatty acid utilization and changes in lipid profiles. In contrast, higher dissolved oxygen levels increase oxygen availability, boosting fatty acid oxidation and utilization, leading to more effective lipid metabolism and better overall nutritional quality (Yang et al., 2019). Likewise, Thulasi et al. (2020) explained that salinity (˂15 ppt) facilitates optimal aerobic respiration and improves the production of essential fatty acids. Similarly, Shin et al. (2023) reported that *Lates calcarifer* supplemented with *Lactobacillus plantarum* at a salinity of 12–35 ppt and 3.1–6.8 mg/L dissolved oxygen levels, showed varied levels of EPA and DHA, i.e., 1.19–0.88 and 0.63–1.08 g/100 g, respectively. Higher salinity (i.e., >35 ppt) is linked with cellular dehydration, which consequently reduces the synthesis of ploy-unsaturated fatty acids like EPA and DHA (Mahdhi et al., 2020). In this nexus, Butt et al. (2021) showed that the inoculation of *Penaeus vannamei* with *Lactobacillus rhamnosus* at salinity of 10 ppt and dissolved oxygen of 6.5 mg/L resulted in significantly higher levels of DHA and EPA, i.e., 1.2 and 1.05 g/100 g, followed by lower concentrations i.e., 0.72 and 0.43 g/100 g at low dissolved oxygen of 2 mg/L and higher salinity of 28 ppt. Likewise, Xie et al. (2019) explained that exposure of L. *vannamei* to *Lactobacillus rhamnosus,* 5 ppt salinity and 8 mg/L of dissolved oxygen delineated higher levels of EPA and DHA i.e., 1.45 and 1.12 compared with the freshwater L. *vannamei* i.e., 0.88 and 0.6 g/100 g, respectively. High dissolved oxygen (˃7 mg/L) increased level of enolase enzyme for the biosynthesis of ployunsaturated fatty acids e.g., EPA and DHA (Madhana et al., 2021).

**Table 5:** Fatty acids profile of L. *vannamei* meat and head (g/100 g).

Fatty acids	Huang meat	Wei meat	T3BS meat	Huang head	Wei head	T3BS head
Saturated Fatty Acids (SFAs)						
C12:0	0.02 ± 0.00^d^	0.01 ± 0.00^d^	0.01 ± 0.00^d^	0.12 ± 0.01^b^	0.05 ± 0.01^c^	0.19 ± 0.03^a^
C13:0	0.09 ± 0.01^a^	0.06 ± 0.01^bc^	0.07 ± 0.01^b^	0.04 ± 0.01^c^	0.07 ± 0.01^b^	0.08 ± 0.02^a^
C14:0	1.12 ± 0.09^d^	1.10 ± 0.06^d^	1.11 ± 0.08^d^	9.28 ± 0.21^a^	4.49 ± 0.18^c^	6.55 ± 0.17^b^
C15:0	1.10 ± 0.04^d^	1.05 ± 0.03^d^	1.02 ± 0.02^de^	1.77 ± 0.05^c^	2.41 ± 0.07^b^	3.12 ± 0.09^a^
C16:0	2.21 ± 0.07^c^	1.19 ± 0.02^d^	2.21 ± 0.06^c^	16.9 ± 0.18^b^	31.86 ± 0.21^a^	30.05 ± 0.23^a^
C17:0	0.97 ± 0.02^c^	0.91 ± 0.03^c^	0.96 ± 0.04^c^	2.80 ± 0.06^b^	2.57 ± 0.07^bc^	4.49 ± 0.09^a^
C18:0	1.80 ± 0.03^d^	1.76 ± 0.05^d^	1.73 ± 0.04^d^	25.94 ± 0.11^a^	14.95 ± 0.22^c^	23.54 ± 0.018^b^
C20:0	0.36 ± 0.02^d^	0.29 ± 0.02^e^	0.34 ± 0.03^d^	0.91 ± 0.04^c^	1.08 ± 0.06^a^	1.03 ± 0.05^b^
C21:0	0.03 ± 0.01^c^	0.04 ± 0.01^c^	0.03 ± 0.010^c^	0.17 ± 0.02^b^	0.52 ± 0.04^a^	0.20 ± 0.02^b^
C22:0	0.09 ± 0.01^d^	0.07 ± 0.01^d^	0.10 ± 0.01^d^	0.83 ± 0.05^b^	1.44 ± 0.09^a^	0.59 ± 0.16^c^
C23:0	0.92 ± 0.04^d^	0.89 ± 0.05^d^	0.90 ± 0.03^d^	2.03 ± 0.17^b^	1.13 ± 0.08^c^	3.11 ± 0.21^a^
C24:0	0.05 ± 0.01^c^	0.03 ± 0.01^c^	0.05 ± 0.01^c^	0.74 ± 0.08^a^	0.72 ± 0.06^a^	0.24 ± 0.03^b^
Monounsaturated Fatty Acids (MUFAs)						
C16:1	0.20 ± 0.01^d^	0.19 ± 0.02^d^	0.16 ± 0.01^d^	3.41 ± 0.08^b^	2.29 ± 0.06^c^	3.64 ± 0.09^a^
C17:1	0.01 ± 0.00^d^	0.02 ± 0.00^d^	0.01 ± 0.00^d^	0.20 ± 0.02^c^	0.24 ± 0.03^b^	0.52 ± 0.05^a^
C18:1	0.77 ± 0.04^d^	0.57 ± 0.03^de^	0.71 ± 0.05^d^	3.30 ± 0.15^c^	12.67 ± 0.11^b^	5.72 ± 0.22^a^
C20:1	0.05 ± 0.01^c^	0.03 ± 0.01^c^	0.04 ± 0.01^c^	0.71 ± 0.06^b^	1.21 ± 0.12^a^	0.61 ± 0.04^bc^
C22:1	0.01 ± 0.00^d^	0.02 ± 0.00^d^	0.01 ± 0.00^d^	0.12 ± 0.02^b^	0.19 ± 0.02^a^	0.03 ± 0.01^c^
C24:1	0.08 ± 0.02^c^	0.09 ± 0.02^c^	0.07 ± 0.01^cd^	0.34 ± 0.06^a^	0.24 ± 0.04^b^	0.05 ± 0.01^d^
Polyunsaturated Fatty Acids (PUFAs)						
C18:2n-6	0.03 ± 0.01^c^	0.01 ± 0.00^c^	0.02 ± 0.00^c^	11.05 ± 0.14^a^	10.82 ± 0.22^ab^	0.83 ± 0.04^b^
C18:3n-3	0.09 ± 0.02^a^	0.03 ± 0.00^b^	0.04 ± 0.01^b^	0.03 ± 0.00^b^	0.01 ± 0.00^c^	0.02 ± 0.00^bc^
C18:3n-6	0.01 ± 0.00^c^	0.03 ± 0.01^c^	0.05 ± 0.01^c^	0.75 ± 0.07^a^	0.74 ± 0.06^a^	0.36 ± 0.04^b^
C20:2n-6	0.79 ± 0.07^b^	0.77 ± 0.06^b^	0.59 ± 0.04^d^	0.64 ± 0.06^c^	0.96 ± 0.08^a^	0.57 ± 0.03^d^
C20:3n-3	0.02 ± 0.00^c^	0.02 ± 0.00^c^	0.01 ± 0.00^c^	0.11 ± 0.02^a^	0.08 ± 0.01^b^	0.13 ± 0.02^a^
C20:3n-6	0.99 ± 0.04^a^	0.92 ± 0.05^b^	0.90 ± 0.03^b^	0.12 ± 0.01^c^	0.15 ± 0.02^c^	0.10 ± 0.01^c^
C20:4n-6	0.6 ± 0.01^c^	0.03 ± 0.01^cd^	0.05 ± 0.01^c^	0.14 ± 0.02^b^	0.70 ± 0.04^a^	0.05 ± 0.01^c^
C20:5n-3 (EPA)	1.11 ± 0.05^d^	1.10 ± 0.08^d^	1.09 ± 0.06^d^	9.86 ± 0.23^b^	3.09 ± 0.12^c^	7.24 ± 0.28^a^
C22:2n-6	0.03 ± 0.01^c^	0.02 ± 0.00^c^	0.01 ± 0.00^c^	0.09 ± 0.02^a^	0.06 ± 0.01^b^	0.05 ± 0.01^b^
C22:4n-6	0.14 ± 0.02^d^	0.12 ± 0.01^d^	0.13 ± 0.02^d^	0.93 ± 0.06^b^	0.24 ± 0.03^c^	1.61 ± 0.12^a^
C22:5n-3	0.01 ± 0.00^bc^	0.01 ± 0.00^bc^	0.01 ± 0.00^bc^	0.04 ± 0.01^b^	0.03 ± 0.00^b^	0.91 ± 0.06^a^
C22:5n-6	0.04 ± 0.01^c^	0.03 ± 0.01^c^	0.03 ± 0.01^c^	0.14 ± 0.02^a^	0.08 ± 0.01^b^	0.13 ± 0.02^a^
C22:6n-3 (DHA)	0.79 ± 0.03^d^	0.71 ± 0.02^de^	0.69 ± 0.04^e^	6.45 ± 0.18^a^	4.91 ± 0.11^b^	4.34 ± 0.09^c^

Results are present as means ± SD and n = 3. Values with different alphabets are significantly (*p* *<* *0.05*) different among rows.

## 4 Conclusion

*L. lactis* D1813, with the favorable environmental conditions of salinity and higher dissolved oxygen, significantly improved the nutritional profile of L. *vannamei*. Further, among the study groups of L. *vannamei*, the Huang meat group revealed higher levels of essential minerals, e.g., K, Mg, P, Cu, Se, Fe, and Zn. Also, the highest levels of astaxanthin in the Huang head group elucidate the nutritional potential of L. *vannamei* head in food value addition. The Huang meat group also showed greater chewiness and cohesiveness, suggesting enhanced textural qualities essential for consumer acceptance. Furthermore, the highest concentration of amino acids such as lysine, phenylalanine, leucine, isoleucine, valine, threonine, methionine, and arginine in Huang meat group indicates the nutritional potential of the Huang meat group in improving health. Additionally, the highest level of ploy-unsaturated fatty acids, such as eicosapentaenoic acid and docosahexaenoic acid, observed in the Huang head group, exhibits its ability to prevent several health challenges. Conclusively, using L. *lactis* D1813 at 10^6^ CFU/mL with optimum salinity (i.e., 8 ppt) and dissolved oxygen (i.e., 8.5 mg/L) significantly improved the nutritional profile in L. *vannamei*. Further, studies could be conducted to assess the probiotic effect on shrimp physiology, immune response, and disease resistance.

## CRediT authorship contribution statement

**Muhammad Adil:** Writing – review & editing, Writing – original draft, Resources, Project administration, Methodology, Investigation, Data curation, Conceptualization. **Guo Xinbo:** Writing – review & editing, Validation, Supervision, Project administration, Methodology, Investigation, Conceptualization. **Junpeng Cai:** Writing – review & editing, Validation, Supervision, Project administration, Methodology, Investigation, Conceptualization. **Muhammad Waseem:** Writing – review & editing, Writing – original draft, Visualization, Validation, Software, Formal analysis. **Muhammad Faisal Manzoor:** Writing – review & editing, Visualization, Validation, Software, Investigation, Writing – original draft. **Crossby Osei Tutu:** Writing – review & editing, Visualization, Validation, Software, Formal analysis, Investigation.

## Declaration of competing interest

The authors declare that they have no known competing financial interests or personal relationships that could have appeared to influence the work reported in this paper.

## Data Availability

The data associated with this study are included in article/supp. material/referenced in article.
